# The effects of pomegranate extract on blood flow, vessel diameter, and exercise tolerance

**DOI:** 10.1186/1550-2783-11-S1-P4

**Published:** 2014-12-01

**Authors:** Malia N Melvin, Eric T Trexler, Erica J Roelofs, Hailee L Wingfield, Abbie E Smith-Ryan

**Affiliations:** 1 Applied Physiology Laboratory, The University of North Carolina, Chapel Hill, North Carolina, USA

## Background

During exercise, the demand for oxygen and energy substrates is elevated in active skeletal muscle. Supplements with high nitrate and polyphenol content have been shown to increase nitric oxide production and enhance exercise efficiency. The current randomized, double-blind, placebo-controlled, crossover study aimed to investigate the acute effects of pomegranate extract on blood flow, vessel diameter, and exercise performance in active individuals.

## Methods

Nineteen men and women (Mean ± SD; Age: 22.2 ± 2.2 yrs; Height: 174.8 ± 10.7 cm; Body mass: 71.9 ± 13.5 kg) performed a graded exercise treadmill test to volitional exhaustion to determine maximal oxygen consumption and peak velocity (PV). Treatment order was randomly assigned. Participants returned after 24-48 hours, and ingested two, 500mg capsules of either placebo (PL; 95% maltodextrin, 5% purple carrot and hibiscus for color) or pomegranate extract (PE; 1000 mg, TRUE Pomegranate Extract [NITRO_2_GRANIT^™^], Stiebs Nature Elevated, Madera, CA) with 6 oz. of water. Brachial artery blood flow was assessed using a GE logiq-e B-mode ultrasound (GE Healthcare, Wisconsin, USA) at baseline and 30 minutes post ingestion (30PI). In random order, subjects performed three treadmill runs to exhaustion at 90%, 100%, and 110%PV to determine critical velocity (CV), anaerobic running capacity (ARC), and time to exhaustion (TTE). Blood flow was assessed immediately after and 30 minutes after each exercise bout. A vitality scale was completed 30PI of PE or PL; a visual analog (pain) scale (VAS) was completed 30PI and immediately post exercise. After a 7-10 day washout period, participants repeated the same procedures, ingesting the opposite supplement. Separate mixed factorial ANOVAs were performed for blood flow, vessel diameter, CV, ARC, TTE, VAS, and vitality scale questions. Consent to publish the results was obtained from all participants.

## Results

Blood flow was significantly augmented 30PI of PE in comparison to PL (p=0.033). Vessel diameter was significantly larger following 30 minutes post exercise with PE (p=0.036). Ingestion of the PE was found to significantly augment TTE at 90%PV (p=0.009) and 100% PV (p=0.027). On the vitality scale, the following statement, “At this moment I feel alive and vital” was found to be significantly greater 30PI of PE compared to PL (p=0.037).

## Conclusions

Acute ingestion of PE 30 minutes prior to exercise may enhance vessel diameter, blood flow, and improve exercise tolerance. Results of the current study indicate that PE is an ergogenic aid for submaximal running, eliciting beneficial effects on blood flow.

